# Ecological niche modelling and predicted geographic distribution of *Lutzomyia cruzi*, vector of *Leishmania infantum* in South America

**DOI:** 10.1371/journal.pntd.0006684

**Published:** 2018-07-30

**Authors:** Everton Falcão de Oliveira, Eunice Aparecida Bianchi Galati, Alessandra Gutierrez de Oliveira, Elizabeth Ferreira Rangel, Bruno Moreira de Carvalho

**Affiliations:** 1 Instituto Integrado de Saúde, Universidade Federal de Mato Grosso do Sul, Campo Grande, MS, Brasil; 2 Departamento de Epidemiologia, Faculdade de Saúde Pública, Universidade de São Paulo, São Paulo, SP, Brasil; 3 Instituto de Biociências, Universidade Federal de Mato Grosso do Sul, Campo Grande, MS, Brasil; 4 Laboratório Interdisciplinar de Vigilância Entomológica em Diptera e Hemiptera, Instituto Oswaldo Cruz, Fundação Oswaldo Cruz, Rio de Janeiro, RJ, Brasil; Fundacao Oswaldo Cruz, BRAZIL

## Abstract

In some transmission foci of *Leishmania infantum* in Brazil, *Lutzomyia cruzi* could be considered the main vector of this pathogen. In addition, *L*. *cruzi* is a permissive vector of *L*. *amazonensis*. Its geographical distribution seems to be restricted and limited to *Cerrado* and Pantanal biomes, which includes some areas in Brazil and Bolivia. Considering that predicting the distribution of the species involved in transmission cycles is an effective approach for assessing human disease risk, this study aims to predict the spatial distribution of *L*. *cruzi* using a multiscale ecological niche model based in both climate and habitat variables. Ecological niche modelling was used to identify areas in South America that are environmentally suitable for this particular vector species, but its presence is not recorded. Vector occurrence records were compiled from the literature, museum collections and Brazilian Health Departments. Bioclimatic variables, altitude, and land use and cover were used as predictors in five ecological niche model algorithms: BIOCLIM, generalised linear model (logistic regression), maximum entropy, random forests, and support vector machines. The vector occurs in areas where annual mean temperature values range from 21.76°C to 26.58°C, and annual total precipitation varies from 1005 mm and 2048 mm. Urban areas were most present around capture locations. The potential distribution area of *L*. *cruzi* according to the final ecological niche model spans Brazil and Bolivia in patches of suitable habitats inside a larger climatically favourable area. The bigger portion of this suitable area is located at Brazilian States of Mato Grosso do Sul and Mato Grosso. Our findings identified environmentally suitable areas for *L*. *cruzi* in regions without its known occurrence, so further field sampling of sand flies is recommended, especially in southern Goiás State, Mato Grosso do Sul (borders with Mato Grosso, São Paulo and Minas Gerais); and in Bolivian departments Santa Cruz and El Beni.

## Author summary

Leishmaniases are vector-borne diseases caused by *Leishmania* parasites which are transmitted to humans by the bites of infected female sand flies. The sand fly *Lutzomyia cruzi* is the vector of *Leishmania infantum*, the causative agent of visceral leishmaniasis (VL), in some specific areas of Brazil. The transmission of *Leishmania* species is climate-sensitive and involves complex ecological interactions between parasites, vectors and hosts. Considering that the vectors are strongly sensitive to climatic and environmental conditions, studies of their geographical distribution are important for understanding the eco-epidemiology of VL, as well as for the planning of disease control actions. The ecological niche of a species is a critical determinant of its distribution. Therefore, we conducted a study to evaluate and model the ecological niche of *L*. *cruzi* and predict susceptible areas to its occurrence in South America. The potential distribution area of *L*. *cruzi* according to the final ecological niche model spans Brazil and Bolivia in patches of suitable habitats inside climatically favourable areas. *Cerrado* and Pantanal biomes comprise the biggest portion of this suitable area which includes three Brazilians states, and some areas in Bolivia. Our findings reinforce the importance of conducting more ecological studies on sand fly fauna.

## Introduction

World Health Organization data show that vector-borne diseases represent more than 17% of the global burden of all infectious diseases, causing more than 1 million deaths per year [1]. The dynamics and intensity of transmission of pathogens exhibit significant spatial and temporal heterogeneity, especially in vector-borne diseases [2,3]. Part of this lies in the fact that vector-borne diseases are climate-sensitive, because the species involved in their complex cycles of transmission are highly dependent on climatic variables [4–6]. In addition, there is evidence that ongoing climate change is affecting, and will continue to affect the distributions and burdens of these infections [4].

Predicting the distribution of the species involved in transmission cycles is an effective approach for assessing human disease risk. The spatial distribution of a species is a reflection of its ecology and evolutionary history, influenced by specific factors depending on the spatial scale [7–9]. Species distributions are hierarchically structured in space, with climatic variables limiting distributions at coarse scales, habitat variables gaining importance as the scale narrows, and biotic interactions affecting distributions at microscales [9,10].

Leishmaniases are climate-sensitive diseases transmitted to humans by the bites of female sand flies (Diptera: Psychodidae) infected with *Leishmania* parasites. The distribution and behaviour of the species involved in the transmission cycle, especially of the sand fly vectors, are strongly affected by climatic variables, such as precipitation, temperature and humidity [11,12]. In Latin America, *Lutzomyia longipalpis* is the main vector of *Leishmania infantum*, the causative agent of visceral leishmaniasis (VL) [13,14]. Due to its great epidemiological importance and wide distribution, *L*. *longipalpis* has been the object of different studies on the effects of environmental variables and anthropogenic environmental changes on its ecology [15–20]. Some of these studies have used ecological niche modelling to estimate the geographic distribution of this vector and predict its expansion or contraction under climate change scenarios [18–20].

However, in some transmission foci of *L*. *infantum* in Brazil, the sand fly *L*. *cruzi* may be acting as the main vector of this protozoan due to absence of *L*. *longipalpis* [21–25]. Although there were suspicions that *L*. *cruzi* was the vector responsible for the transmission of *L*. *infantum* since the 1980s [21,22], only recently this phlebotomine sand fly was confirmed as a proven vector of *L*. *infantum* [25], based on the Killick-Kendrick criteria [26], and as a permissive vector of *L*. *amazonensis* [25]. *Lutzomyia cruzi* can also act as an alternative vector in the location where both sand flies occur in sympatry [19]. In Brazil, the geographical distribution of *L*. *cruzi* seems to be restricted and limited to *Cerrado* and Pantanal biomes [23,24,27–29]. There are also reports of the presence of *L*. *cruzi* in Bolivia [30]. Recent evidences suggest introgressive hybridization between *L*. *cruzi* and *L*. *longipalpis* based on molecular analyses [31,32], reinforcing the idea that they are sibling species.

Even though *L*. *cruzi* has medical and epidemiological relevance, until now there are few published reports focused on the ecology and effects of environmental variables on the distribution and abundance of this sand fly [19,21,27,28,33,34]. A recent study applied ecological niche models to predict the distributions of *L*. *longipalpis* and *L*. *cruzi* in Brazil, but models were based on both species together, thus making it impossible to evaluate their distributions separately [19]. A further assessment of the potential distribution of *L*. *cruzi* is needed, especially for those areas where *L*. *longipalpis* does not occur.

Considering that ecological niche modelling represents a tool for monitoring disease trends in natural ecosystems and identify opportunities to mitigate the impacts of climate-driven disease emergence [35], this report aims to predict the spatial distribution of *L*. *cruzi* using a multiscale ecological niche model based in both climate and habitat variables. Besides contributing to the study of the ecological niche of *L*. *cruzi*, our goal includes the identification of specific areas in Brazil and neighbour countries that are environmentally suitable for this particular vector species, but its presence is not recorded.

## Methods

### Occurrence records

We conducted a literature review to compile records of the presence of *L*. *cruzi*. On July 2016, the online databases PubMed, ISI, Scopus and SciElo were searched for relevant studies using the terms ‘*Psychodidae*’ and ‘*Lutzomyia*’. After removal of duplicate references, the papers were scanned for mention of *L*. *cruzi* captures, and all records compiled in a Microsoft Excel database with the available description of the capture sites (country, state/province/department, district/municipality, and locality). Additionally, the sand fly distribution lists compiled by Martins et al. [36], Young & Duncan [37], Aguiar & Medeiros [38] and Galati [39] were also consulted to ensure known presence records were not missed. As females of *L*. *cruzi* and *L*. *longipalpis* are morphologically indistinguishable [37,39], only the records with species identification based on captured males were considered as valid.

The main sand fly collections in Brazil were physically visited to search for additional unpublished records of the species. These included Centro de Pesquisas René Rachou (FIOCRUZ, Belo Horizonte, assisted by Dr J. D. Andrade-Filho), Instituto Butantan (IBUT, São Paulo, assisted by Dr R. Moraes), Instituto Evandro Chagas (IEC, Belém, assisted by Dr T. Vasconcelos dos Santos), Instituto Oswaldo Cruz (FIOCRUZ, Rio de Janeiro, assisted by Dr J. M. Costa), Instituto de Pesquisas da Amazônia (INPA, Manaus, assisted by Dr R. Freitas and Dr M. L. Oliveira), Universidade de São Paulo/Faculdade de Saúde Pública (USP, São Paulo, assisted by Prof. M. A. Sallum), and Universidade de São Paulo/Museu de Zoologia (USP, São Paulo, data provided by Dr A. J. Andrade). The online databases SpeciesLink (http://splink.cria.org.br/) and GBIF (https://www.gbif.org/) were also searched for presence records on February 2018.

All presence records were associated with geographical coordinates (latitude and longitude) and classified in three levels according to their spatial precision: High level: coordinates of the capture site were available in the original source of the record; Medium level: coordinates were obtained at Google Earth (https://earth.google.com/) by visually searching for the capture site when its description was available in the source of the record; Low level: coordinates of the municipality/district centre were obtained at Google Earth when the source of the record had no information on the capture locality, but only at this administrative level. We excluded from the database those records with information only at state/province/department or country levels.

The occurrence database thus contained the following information for each record: country, state/province/department, municipality/district, locality, year of capture, longitude, latitude, spatial precision, reference (S1 Table). The year of capture and spatial precision were used to split the records in separate sets for model training and validation, in accordance with the spatial and temporal precision of the variables used in the ecological niche models.

### Pseudo-absence records

As some modelling algorithms require presence/absence data, we randomly sampled pseudo-absences in the space outside the environmental domain favourable for the species [40] but restricted to a maximum distance of 1000 km from the presence records. This environmental domain was estimated using the bioclimatic envelope model BIOCLIM [41]. The number of pseudo-absences was 10 times the number of presence records for each model run. We ran the pseudo-absence sampling procedure once for each modelling step (climate and habitat models). These procedures were performed in R platform [42], using the packages *raster* [43] and *dismo* [44].

### Climate and habitat variables

We obtained historical (1970–2000) climate data for South America at WorldClim (version 2), an online database of 19 bioclimatic variables derived from monthly averages of temperature and precipitation [45]. For the climate model, we obtained the variables at the spatial resolution of 2.5 minutes (approximately 5x5km per pixel), which is an adequate coarse resolution where climate influences species distributions [9]. We selected a subset of the original 19 variables by running a Pearson correlation matrix and retaining only the six less correlated ones (*r* < 0.6). The final set of climate variables used to run the climate model consisted of annual mean temperature (BIO1), mean diurnal range of temperature (BIO2), temperature seasonality (BIO4), annual precipitation (BIO12), precipitation seasonality (BIO15) and precipitation of warmest quarter (BIO18) [45].

Remote sensing variables representing vegetation and topography were used as potential habitat indicators of *L*. *cruzi*. The Enhanced Vegetation Index (EVI), a product of the MODIS (Moderate Resolution Imaging Spectroradiometer) sensor was obtained at NASA’s EarthExplorer website (https://earthexplorer.usgs.gov/) and processed with the MODIS Reproject Tool (https://lpdaac.usgs.gov). Monthly EVI data for 2000–2015 was obtained for the study area at the spatial resolution of 1 km. A Principal Component Analysis (PCA) was performed in order to reduce collinearity in the dataset. We retained the first five components, because they represented 99% of the cumulative variance in the monthly EVI dataset. Altitude, aspect and slope variables were derived from a digital elevation model from SRTM (Shuttle Radar Topographic Mission) and obtained at AMBDATA, an online database of environmental layers maintained by INPE (Instituto Nacional de Pesquisas Espaciais, http://www.dpi.inpe.br/Ambdata/). The eight habitat variables were resampled to 1 km^2^ resolution by bilinear interpolation and cropped at the extension of the study area, which was determined by the results of the climate model. All variable processing was done using the R packages *raster* and *RSToolbox* [46].

### Ecological niche description

To describe the ecological niche of *L*. *cruzi*, the values of the main bioclimatic variables and altitude in the location of each presence record were extracted. We also assessed the types of land use and cover where the vector occurs using data from MapBiomas (http://mapbiomas.org/), a high-resolution database of annual land use and cover for Brazil. Each presence record was associated with the land use and cover data of the same year of capture. We excluded the records with low spatial precision at this step, because they do not match the native resolution of the MapBiomas data layers (30x30m). The percentage of each land cover type was extracted in a 500 m buffer created on each presence record. Analyses were performed in R package *raster*.

### Ecological niche modelling

There are several algorithms available for developing ecological niche models, which produce different results and predictive maps even when running with the exact same input data [47–49]. There is not a consensus on the literature about one single best algorithm, thus researchers are encouraged to apply different methods to overcome this methodological uncertainty in their model predictions [50,51]. Therefore, we applied the same five modelling algorithms as McIntyre et al. [52], which had satisfactory results in niche models of Brazilian sand flies: BIOCLIM, Generalised Linear Models (GLM, logistic regression), Maximum Entropy (MaxEnt), Random Forests (RANFOR), and Support Vector Machines (SVM). For a short description of the five algorithms, see McIntyre et al. [52].

To reduce spatial auto-correlation, we randomly selected a subset of species occurrences which were at least 10 km apart from the nearest record. We ran all models with their default settings on the *dismo* package of R platform. In order to use the whole set of unique presence/pseudo-absence records in model training, we used 10-fold cross-validation, with 10% of the records retained for internal model testing. For internal evaluation, we used the True Skill Statistic (TSS), which ranges from -1 to +1, with +1 indicating complete agreement between predicted and observed records, and values close to and below 0 representing models no better than random predictions [53]. Model outputs with TSS scores lower than 0.6 were discarded. Outputs with the highest TSS scores from each algorithm were overlaid and consensus areas extracted by the majority ensemble rule [54]. Final maps were produced based on the consensus between the five modelling algorithms. Uncertainty was mapped by calculating the standard deviation of pixel values from model outputs produced by each of the five algorithms.

Because of the great difference in spatial precision of the species records, we ran two models with adequate settings for each spatial scale (Table 1). On a first step, we ran a climatic suitability model at the coarse spatial resolution of the climatic variables (2.5 minutes). For this model we used the set of *L*. *cruzi* records captured between 1970 and 2000 with the six bioclimatic variables. Model calibration area was restricted to a hypothesised accessible area of 1000 km around all known species records [55]. As we were aiming for a more conservative output for this first model, we chose the “zero omission” threshold rule [56] to convert model outputs into binary predictions. With this threshold rule, all presence records are retained inside the predicted area of occurrence, thus maximizing sensitivity (the proportion of correctly predicted presences), but sacrificing specificity (the proportion of correctly predicted absences).

**Table 1:** Summary of model settings.

	Climatic Suitability model	Habitat Suitability model
**Species records for model train**	Spatial precision: High, Medium, Low Years of capture: 1970–2000 N = 52	Spatial precision: High, Medium Years of capture: 2004–2013 N = 22
**Model calibration area (M)**	1000 km buffer around all presence records	100 km buffer around climatically suitable area
**Variables**	Annual mean temperature (BIO1) Mean diurnal range of temperature (BIO2), Temperature seasonality (BIO4) Annual precipitation (BIO12) Precipitation seasonality (BIO15) Precipitation of warmest quarter (BIO18)	Enhanced Vegetation Index (5 principal components) Altitude Slope Aspect
**Spatial resolution**	2.5 minutes	0.5 minute
**Threshold rule**	Zero omission	Maximum training sensitivity and specificity
**Species records for model validation**	Spatial precision: High, Medium, Low Years of capture: 2003–2013 N = 25	Spatial precision: High, Medium Years of capture: 1970–2000 N = 7

The resulting binary map of climatic suitability was then used to limit the calibration area of the habitat suitability model, which was based on the vegetation and topography variables at higher scale (Table 1). As we narrowed the spatial resolution, at this second stage we only used the presence records classified as precision levels high and medium, with capture years matching the variables (2004–2013). The same model settings were applied, except for the threshold rule to produce binary predictions. For the final models, we chose threshold values that maximised both sensitivity and specificity [56]. With this, the final outputs become more objective, minimising both false positives and false negatives.

External validation of both models was done with independent records, separated from model training (Table 1). Model significance was evaluated by binomial probabilities calculated over binary outputs, and model performance was evaluated by sensitivity (number of correctly predicted presences divided by total number of records). Resulting model outputs were exported to QGIS software version 3.0.1 [57] for preparation of final maps.

## Results

**Fig 1:**
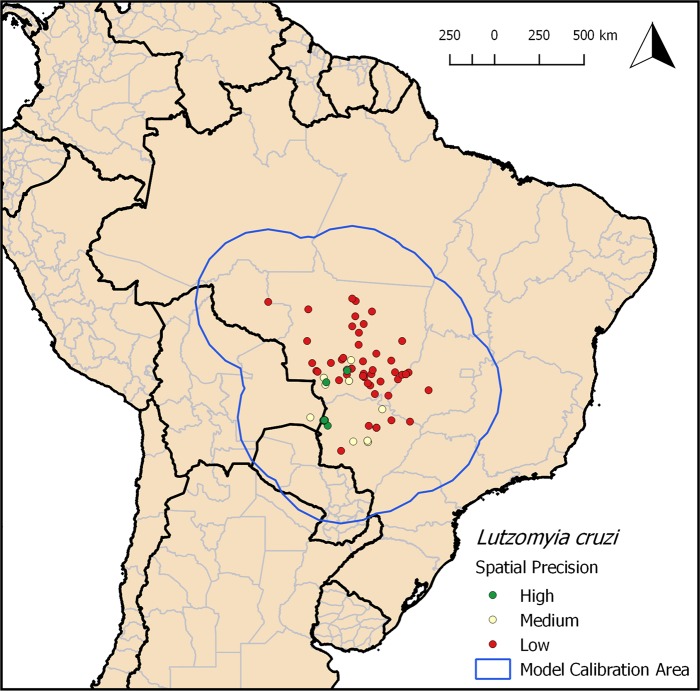
Compiled occurrence records of *Lutzomyia cruzi* classified in different spatial precision levels (green: high; yellow: medium; red: low). The blue line delimits the model calibration area. Map produced in QGIS.

The compiled database included 116 presence records of *L*. *cruzi* with associated geographical coordinates (S1 Table). Most records of the vector are in Mato Grosso and Mato Grosso do Sul Brazilian states, with a single record in State of Goiás and one in Bolivia, in Santa Cruz Department (Fig 1). Most of the records have low spatial precision (68%), followed by records with medium (25%) and high (7%) precision levels (Fig 1).

The vector occurs in areas where annual mean temperature values range from 21.76°C to 26.58°C, and annual total precipitation varies from 1005 mm and 2048 mm (Table 2). In these areas, temperatures in the coldest month of the year reach 11.3°C and the warmest month can reach as high as 34.3°C (Table 2). Extremes of monthly precipitation range from 1 mm to 157 mm (Table 2). In terms of elevation, most records of *L*. *cruzi* occur around 270 m above sea level, with a minimum of 86 m and up to 741 m (Table 2).

**Table 2:** Minimum, median, mean and maximum values of climatic variables and altitude recorded at capture locations of *Lutzomyia cruzi*.

Variable name	Min.	Median	Mean	Max.
Annual Mean Temperature (°C)	21.76	24.91	24.71	26.58
Max Temperature of Warmest Month (°C)	30.3	33.3	33.1	34.3
Min Temperature of Coldest Month (°C)	11.3	15.3	15.13	17.9
Temperature Seasonality (standard deviation *100)	46.77	171.68	164.91	240.74
Annual Precipitation (mm)	1005	1445	1457	2048
Precipitation of Wettest Month (mm)	157	226	242.9	349
Precipitation of Driest Month (mm)	1	17	16.17	41
Precipitation Seasonality (Coefficient of Variation)	51.77	67.54	68.91	83.86
Altitude (m)	86.45	270.84	305.7	741.41

**Fig 2:**
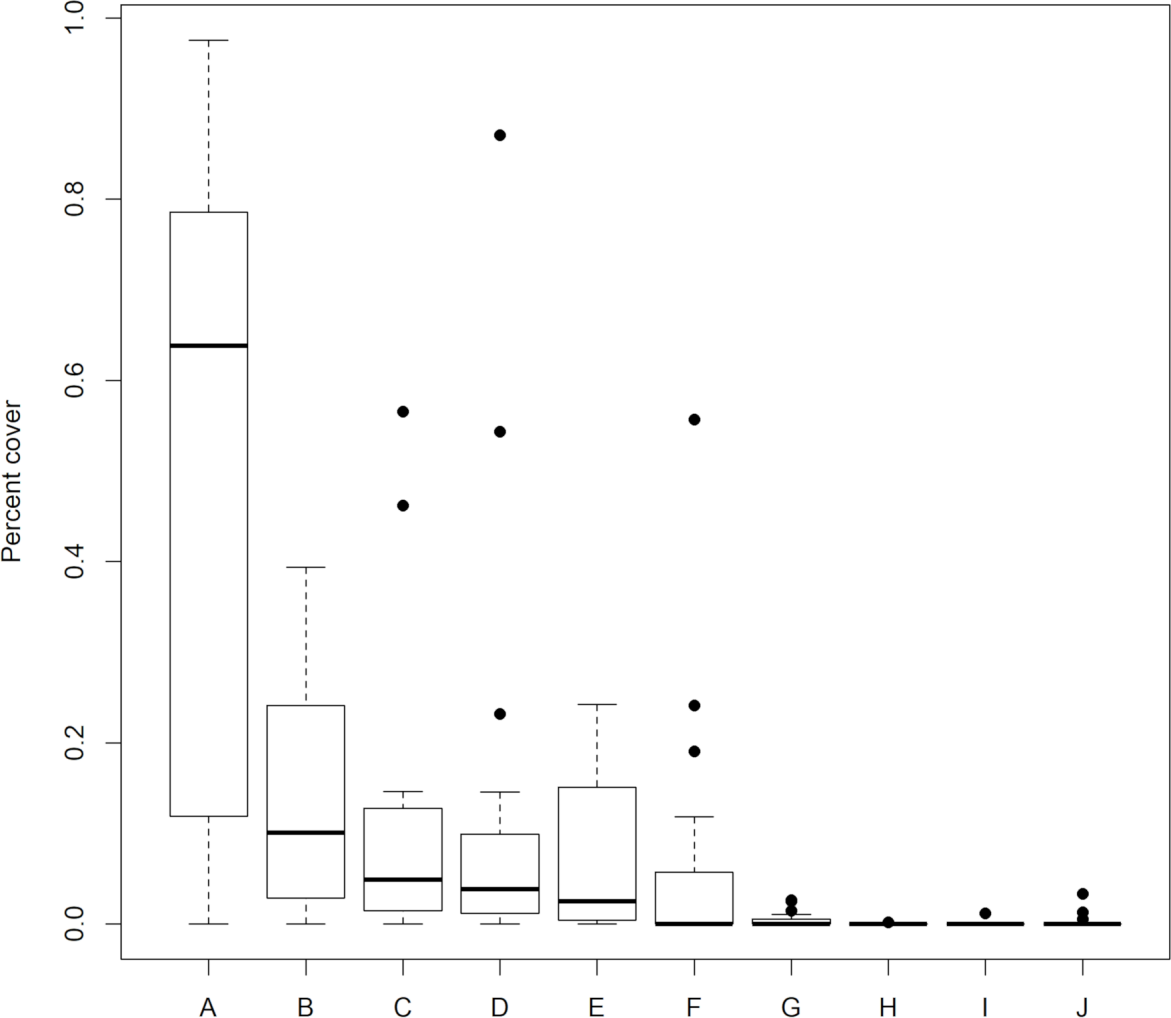
Percentage of land use and cover types observed 500 m around presence records of *Lutzomyia cruzi* in Brazil (N = 20). A) urban area; B) open forest; C) dense forest; D) pasture; E) open field; F) other non-forest formations; G) agriculture or pasture; H) non-forest natural areas; I) water bodies; J) unclassified.

Nine different types of land use and cover were detected around records of *L*. *cruzi* (Fig 2). Urban areas were most present around capture locations (64%), followed by open forests (10%), dense forests (5%), pasture areas (4%), and open fields (3%). The remaining land use and cover types were identified only eventually and are presented in Fig 2.

The TSS scores of the climatic suitability models ranged from 0.48 to 1 (8% were discarded with TSS < 0.6); and in the final models, from 0 to 1 (22% with TSS < 0.6). Outputs produced by different algorithms varied considerably (S1 Fig), but consensus areas showed less uncertainty (S2 Fig). The climatic suitability model performed significantly better than random predictions (binary probabilities, *p* = 0.00498) and had sensitivity of 0.92; while the final ecological niche model was also significant (binary probabilities, *p* < 0.001) with a sensitivity of 0.72.

**Fig 3:**
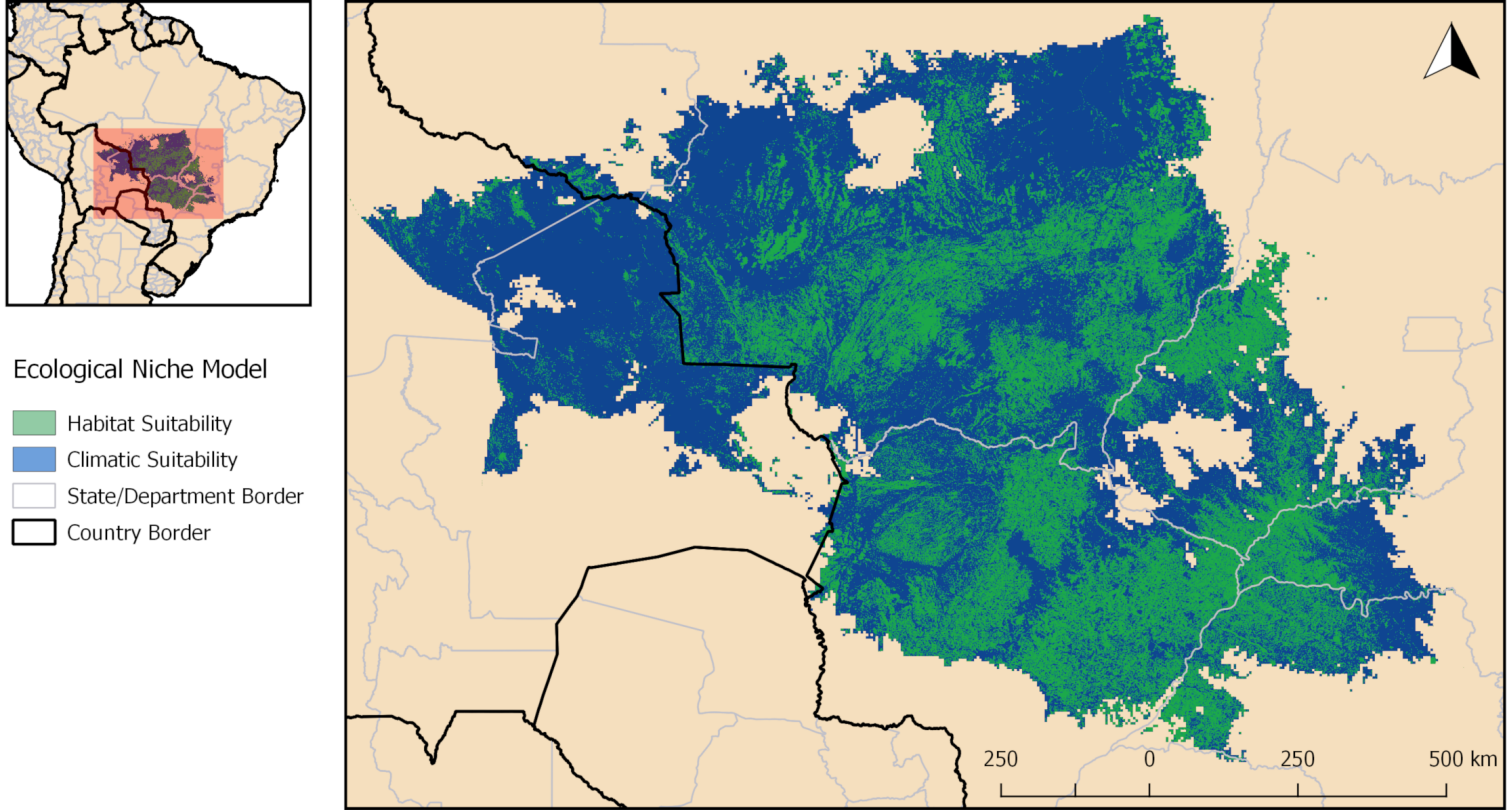
Predicted areas of suitable climate (blue) and habitat (green) for *Lutzomyia cruzi*. Map produced in QGIS.

The coarse resolution model predicted an area of climatic suitability for *L*. *cruzi* that occupies the Central-West region of Brazil, extending westwards into Bolivia (blue and green areas in Fig 3). However, when considering the habitat variables at high resolution, the results of the final ecological niche model show that the area with suitable climate and habitat conditions for *L*. *cruzi* is much smaller, occupying 38.7% of the climatically suitable regions (only green areas in Fig 3).

**Fig 4:**
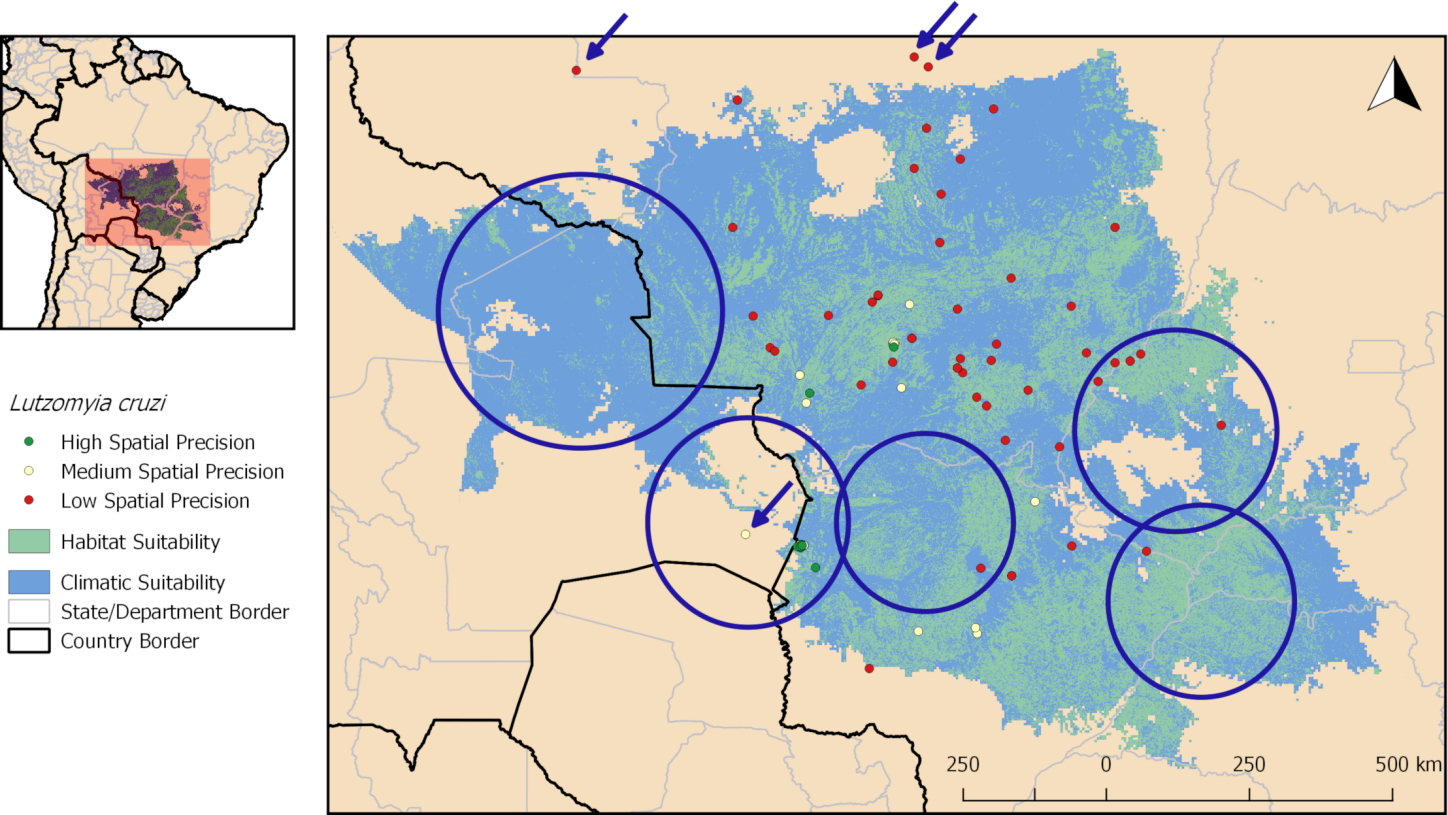
Potential distribution of *Lutzomyia cruzi* based on ecological niche modelling predictions and known presence records. Circles represent areas of environmental suitability that need further field studies to assess vector occurrence. Arrows indicate records that were not predicted by the models. Map produced in QGIS.

The potential distribution area of *L*. *cruzi* according to the final ecological niche model spans Brazil and Bolivia in patches of suitable habitats inside climatically favourable areas. The bigger portion of this suitable area is located at Brazilian States of Mato Grosso do Sul and Mato Grosso, where most known records of the species are located (Fig 4). Four known records of the vector fell out of the predicted area: one in Bolivia (El Carmen), and three in Mato Grosso State (Nova Canaã do Norte, Colíder and Rondolândia) (see arrows in Fig 4). Suitable areas without known occurrence of the vector are located in Bolivian departments Santa Cruz and El Beni; southern State of Goiás in Brazil, as well as northern Mato Grosso do Sul and in border areas with São Paulo and Minas Gerais States (see circles in Fig 4).

## Discussion

This study represents the first report of the predicted spatial distribution of *L*. *cruzi* using a multiscale ecological niche model based on both climate and habitat variables, applying different algorithms for the same data. The final ecological niche model comprises mainly areas of the Central-West region of Brazil and some parts of East Bolivia.

The low number of occurrence records and their low spatial precision were limitations of the modelling process, being the most probable reason for the low TSS scores of a minority of model outputs. We reduced these limitations by discarding outputs with TSS < 0.6 in the final models and subsampling the records by spatial precision, thus running models at appropriate spatial scales. Models produced by different algorithms had great spatial variability, as expected [47–51]. Uncertainty mapping provided more confidence to the areas predicted as environmentally suitable by most algorithms.

Our results describe the ecological niche of *L*. *cruzi* in terms of climate, altitude and vegetation/land cover where the species occurs. The climatic values recorded at capture locations of *L*. *cruzi* are in accordance with the Köppen’s climate classification for most parts of the Central-West region of Brazil: tropical zone with monsoon period (Am) and with dry winter (Aw) [58]. Ecological studies that evaluated the linear relationship between *L*. *cruzi* abundance and climatic variables showed no significant statistical association [24,27,59]. However, it was observed that the species occurs throughout the year, with population peaks in the months with high temperature [21,24,27,59]. These previous studies considered both male and female specimens of *L*. *cruzi* and reported data from regions where *L*. *longipalpis* has not been detected, except in Corumbá city [60]. However, *L*. *longipalpis* was reported in Corumbá only once [60]. Successive sand fly surveys performed by different research groups were unable to confirm the presence of *L*. *longipalpis* in this area [21,27,28,61,62]. It should be noted that the occurrence sites of this vector have annual mean temperature relatively constant and annual precipitation ranging from moderate to high (Table 1).

Altitude data show that most records of *L*. *cruzi* occur in the Central and Southern plateau and in the Pantanal plains of Brazil. This observation allows us to hypothesize that the distribution of *L*. *cruzi* may be limited, among other factors, by altitude, since there is no record of the species in coastal regions. *Cerrado* and Pantanal are the biomes where *L*. *cruzi* mostly occurs, with few observations in southern Amazon. Our results of the percentage of land use and cover types demonstrate that *L*. *cruzi* is present predominantly in urban areas. However, this does not necessarily mean that *L*. *cruzi* prefers urban areas, because most of the sand fly samplings where performed in these areas or in peri-urban localities. Nevertheless, considering that *L*. *cruzi* and *L*. *longipalpis* are sibling species [31,32], the probable preference of *L*. *cruzi* for urbanized environments would not be surprising. As an example, data from the city of Corumbá, State of Mato Grosso do Sul, showed that in the 1980s the greatest abundance of *L*. *cruzi* was in native forest areas with low human interference [21]. Almost 30 years later a lower abundance was observed in the city’s peripheral forests, while in the urban area, the vector increased its abundance [27,28]. Similar situation was found in the city of Camapuã (Fernandes et al., 2017), also located in Mato Grosso do Sul State. No significant association was found between the absolute frequencies of *L*. *cruzi* and percentage of vegetal coverage and three spectral indices (normalized difference vegetation index, NDVI; normalized difference water index, NDWI; impervious surface areas, ISA) [27].

The predicted area of occurrence from our models corroborates a previously published distribution model of *L*. *cruzi* that was restricted to the Central-West region of Brazil [34]. The predicted area of occurrence of *L*. *cruzi* cannot be determined in Andrade-Filho et al. [19], but the general distribution of the species records used in the models is similar. Neither of the two studies give information on the spatial precision of the presence records. Positional uncertainty in species occurrence records have direct effects on ecological niche model predictions [63] and must be considered especially when developing models from secondary data. The vast majority of information available on species occurrence databases from Brazil is restricted to the municipal level. This can lead to serious bias in model predictions, as municipalities in Brazil have widely different areas, ranging from approximately 3 km^2^ to 160,000 km^2^ [64]. It is crucial that the spatial precision of species records match the spatial resolution of the models [65]. With our multiscale approach, we were able to develop models that incorporated the records with low spatial precision, thus reducing positional bias in our predictions. In addition, the spatial thinning process reduced the spatial auto-correlation bias. The four records that were not successfully predicted by the final models had low spatial precision, so it is not possible to determine the exact location of the species occurrence.

Our models predict occurrence areas of *L*. *cruzi* in Bolivia, where the vector was found in chicken coops and pigsties in the town of El Carmen, Santa Cruz District [30]. This is the only published record from the country, and according to our predictions, *L*. *cruzi* is probably present, but so far undetected in many Bolivian regions. Both visceral and cutaneous leishmaniases are endemic in Bolivia with occurrence of *L*. *infantum*, *L*. *braziliensis* and *L*. *amazonensis* [66–69]. However, there are few reports of ecological studies of phlebotomine fauna in this country, so further field sampling of sand flies is recommended.

According to the Brazilian Ministry of Health [70], except for the southwest Minas Gerais State, in the confluence region between the Grande river and the Paranaiba river (boundary with the states of São Paulo, Goiás and Mato Grosso do Sul), there are autochthonous human cases of VL reported in almost all the predicted suitable areas for *L*. *cruzi*. However, in many regions there are also the presence of *L*. *longipalpis* and/or *L*. *cruzi* [19]. A particular region, predicted as favorable to the vector, deserves to be highlighted due to the presence of autochthonous VL cases [70] and absence of *L*. *longipalpis* records according to Andrade et al. [19]: Brazil-Bolivia border in the extreme southwest Rondônia State, in the area adjacent to the municipality of Pimenteiras do Oeste. In Bolivia, few VL human cases have been reported and the disease appears to be restricted to Yungas region in the Beni department [71].

In Brazil, although the vector’s occurrence is widely known in State of Mato Grosso, some municipalities in Mato Grosso do Sul and the southern region of Goiás remain to be investigated. The border region between the states of Minas Gerais, São Paulo and Mato Grosso do Sul is also a predicted area of occurrence according to our models, but without known records of *L*. *cruzi*. This region, where the Paraná river basin divides the states, has many records of *L*. *longipalpis*, especially on the east side of the river [19]. To our knowledge, there is not a published study on the ecological interactions between *L*. *cruzi* and *L*. *longipalpis* that could justify their separation in space. Further studies on the phylogeography of both species might investigate if the Paraná river basin could have been a relevant dispersion barrier for their speciation.

In conclusion, our results contribute to the study of the ecology and distribution of an important vector of VL. The disease is being increasingly reported in urban and peri-urban areas of Brazil, especially because of the geographical expansion of its main vector, *L*. *longipalpis* [72]. Given the genetic proximity of this vector with *L*. *cruzi* [31,32] and its absence in specific VL foci, our predictive maps also indicate potential risk areas of this disease associated with *L*. *cruzi*. It is crucial that entomological surveillance activities are performed in these areas, especially where the vector has not been detected so far.

## Supporting information

S1 Table Compiled presence records of *Lutzomyia cruzi*. (XLSX)

S1 Fig Ecological niche models of *Lutzomyia cruzi* produced by different algorithms. Maps produced in QGIS.
(TIF)

S2 Fig Model uncertainty for climate and habitat models of *Lutzomyia cruzi*. Maps produced in QGIS.
(TIF)
