# Supplementary figures and images for: Ecological niche modelling and predicted geographic distribution of Lutzomyia cruzi, vector of Leishmania infantum in South America
Source: PLoS Negl Trop Dis. 2018 Jul 30;12(7):e0006684. doi: 10.1371/journal.pntd.0006684 (PMC6085070; doi:10.1371/journal.pntd.0006684)

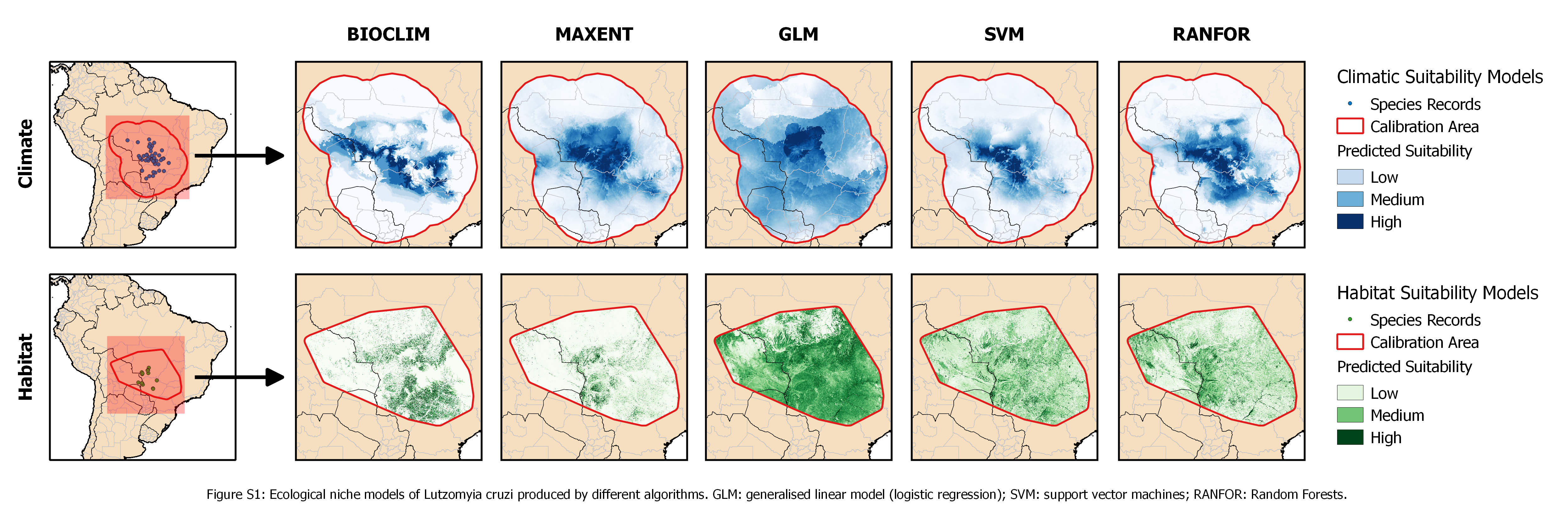

Supplement: S1 Fig — Maps produced in QGIS. (TIF) [file pntd.0006684.s002.tif]

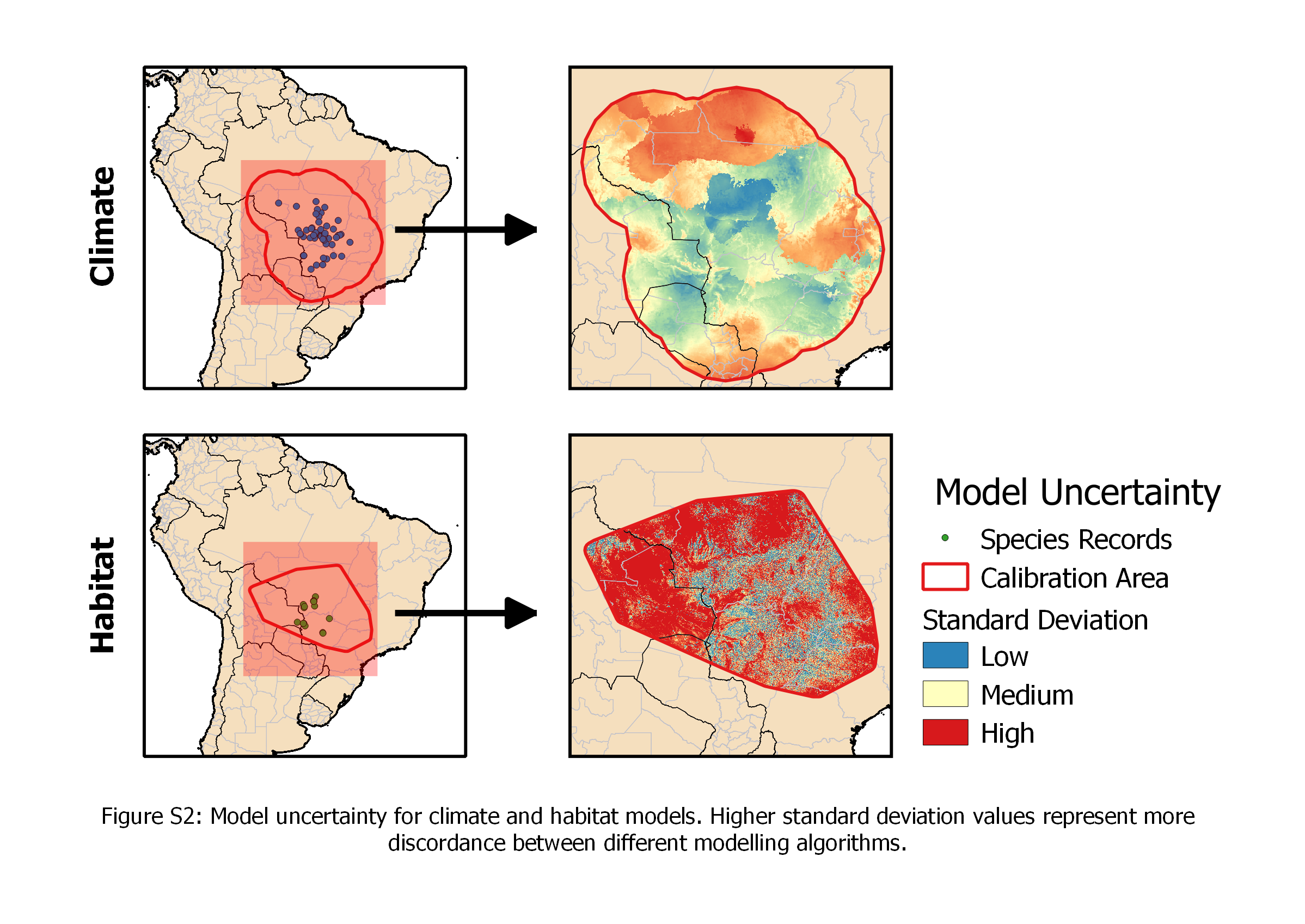

Supplement: S2 Fig — Maps produced in QGIS. (TIF) [file pntd.0006684.s003.tif]
